# Novel insights into transketolase activation by cofactor binding identifies two native species subpopulations

**DOI:** 10.1038/s41598-019-52647-y

**Published:** 2019-11-06

**Authors:** Henry C. Wilkinson, Paul A. Dalby

**Affiliations:** Department of Biochemical Engineering, University College London, London, WC1E 6BT UK

**Keywords:** Holoenzymes, Biological fluorescence, Biocatalysis

## Abstract

Transketolase (TK) cofactor binding has been studied extensively over many years, yet certain mysteries remain, such as a lack of consensus on the cooperativity of thiamine pyrophosphate (TPP) binding into the two active sites, in the presence and absence of the divalent cation, Mg^2+^. Using a novel fluorescence-based assay, we determined directly the dissociation constants and cooperativity of TPP binding and provide the first comprehensive study over a broad range of cofactor concentrations. We confirmed the high-affinity dissociation constants and revealed a dependence of both the affinity and cooperativity of binding on [Mg^2+^], which explained the previous lack of consensus. A second, discrete and previously uncharacterised low-affinity TPP binding-site was also observed, and hence indicated the existence of two forms of TK with high- (TK_high_) and low-affinity (TK_low_). The relative proportions of each dimer were independent of the monomer-dimer transition, as probed by analytical ultracentrifugation at various [TK]. Mass spectrometry revealed that chemical oxidation of TK_low_ led to the formation of TK_high_, which was 22-fold more active than TK_low_. Finally, we propose a two-species model of transketolase activation that describes the interconversions between apo-/holo-TK_high_ and TK_low_, and the potential to significantly improve biocatalytic activity by populating only the most active form.

## Introduction

Transketolase (TK) (EC 2.2.1.1) is a key thiamine pyrophosphate (TPP)-dependent enzyme in the non-oxidative phase of the pentose phosphate pathway (PPP), which branches the glycolytic pathway and diverts metabolic flux through biosynthetic pathways such as pentose sugars and ribose-5-phoshate biosynthesis, a precursor for nucleotide biosynthesis. Another important product of the PPP is the redox cofactor NADPH, an antioxidant that protects against oxidative damage often caused by reactive oxygen species (ROS). The PPP is therefore upregulated during oxidative stress and has several regulatory control points^1–3^, although no such regulation has been characterised previously for transketolase. There is also evidence of upregulation of the PPP in response to other cell stresses, such as osmotic, heat, and heavy metal stress^4,5^.

**Figure 1:**
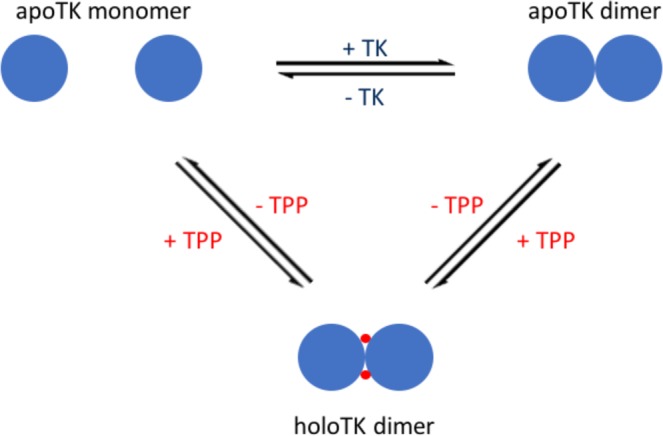
A schematic diagram of the oligomeric states of *E. coli* apo-transketolase (apoTK) and holo-transketolase (holoTK). For simplicity, divalent cations are not shown.

Transketolase catalyses the reversible transfer of a two-carbon ketol group from a donor substrate to an aldose acceptor substrate, forming a new asymmetric C-C bond with high regio- and stereo-specificity. As such reactions remain a significant challenge to synthetic chemists, requiring many steps, and often result in multiple products with lost stereochemistry, biocatalysis with transketolase is also of significant interest to industry^6^. In addition, the reaction can be rendered irreversible, and hence more atom-efficient, using hydroxypyruvate (HPA) as the donor substrate with the concomitant release of CO_2_ as a by-product. Yeast and *E. coli* apo-transketolases exist as monomers that form homodimers at higher protein concentrations^7^. Upon cofactor binding, both the apo-monomer and apo-dimer form a catalytically active homodimer of apparently structurally-identical subunits, with two active sites per homodimer located at the subunit interface (Fig. 1)^8,9^. In its fully active form, each active site is occupied by one molecule of TPP and one divalent cation (M^2+^) such as Ca^2+^, Mg^2+^ or Mn^2+^, meaning each catalytically-active protein homodimer can bind two TPP molecules and two M^2+^ ions. At low concentrations, the inactive apo-transketolase monomer from yeast is activated slowly upon addition of the two cofactors, to form the active homodimer^10,11^. At higher concentrations, cofactor binding to the inactive apo-transketolase dimer leads to the structural organisation of two disordered cofactor-binding loops in the active site, to form the active holo-TK homodimer^11^. The two active sites of several TPP-dependent enzymes have been reported to be non-equivalent in terms of their cofactor affinities^12–14^, substrate binding (half-of-the-sites reactivity)^15–18^ and, in the case of transketolase, their inactivation profiles^7,19,20^. Crystallographic evidence for non-identical active sites has so far been only reported for the E1 component of the pyruvate dehydrogenase complex (PDHc-E1)^21^. The structure highlighted differences in the flexibility of one region near the active site, in each of the two subunits, and described a proton wire between the two TPP-binding sites that potentially mediated their cooperativity in the reaction cycle. However, TK crystal structures obtained to date have not revealed any large structural differences between the two active sites^8,9,22,23^. Nevertheless, the same proton wire could be found, plus a slight difference in the temperature factor (B-factor) distribution between each subunit in the yeast TK crystal structure^22^, which manifested itself as ‘noisier’ or less well-defined coordinates in one subunit. Subsequent re-analysis of the B-factor distribution, along with noted differences in the orientations of Trp391 and Tyr370, which interact to stabilise the cofactor-binding loops, was later interpreted as a slight asymmetrical strain between subunits that may be caused by the one-by-one destabilisation of the holo-sites that leads to a permanent oscillation between two asymmetric states^24,25^.

Transketolase is inactive when bound to only divalent cations, while addition of TPP at relatively high concentrations in the absence of Mg^2+^ can achieve up to 70% activity relative to that in the presence of saturating Mg^2+^ concentrations and non-saturating [TPP]^26,27^. No dissociation constants for TPP have been measured in the absence of Mg^2+^. Addition of Mg^2+^ results in the formation of two active sites that can both bind TPP with a significantly higher affinity relative to that in the absence of Mg^2+^ ^27,28^. The cooperativity of binding when Mg^2+^ is the divalent metal ion is a contentious point with no consensus view. The literature ranges from positive^29^ to negative cooperativity^7^, and then also from active sites with a single dissociation constant^27,28,30–32^, to active sites with two dissociation constants^19^. It is clear, however, that a degree of non-equivalence exists, whether it is in the form of positive or negative TPP-binding cooperativity. While the binding of cofactors to yeast transketolase has been studied extensively, the binding of TPP to *E. coli* transketolase is less well characterised.

The present study was undertaken to directly measure TPP-binding to *E. coli* transketolase in the presence and absence of magnesium ions, using a novel fluorescence-quenching assay. The initial aim was to detect TPP binding directly to provide a much-needed consensus on both the change in dissociation constant, and the binding cooperativity, over a large range of Mg^2+^ and TPP concentrations. Following the unexpected detection of two distinct transketolase species with stark differences in affinity and activity, the origin of the ratio of the two species was probed further, and linked to a specific chemical oxidation, namely single- and double-oxidation of an active-site Cys157 thiol (RSH), to form a sulfenic acid (RSOH) or sulfinic acid (RSO_2_H), respectively.

## Results

### A novel fluorescence quenching-based cofactor-binding assay

The intrinsic fluorescence of transketolase is quenched upon TPP binding^27^ when excited at 280 nm. Our further analysis indicated that TPP has two strong absorption bands at 233 nm and 266 nm and is itself weakly fluorescent when excited at either of these wavelengths. It was therefore decided to excite samples at the lower absorption band (λ_ex_ = 240 nm; λ_em_ = 330 nm). As reported previously for 280 nm excitation, we observed that transketolase fluorescence was quenched upon TPP binding after excitation at 240 nm (Fig. S1, Supplementary Information). The signal generated was corrected for the inner filter effect (IFE), that arises from strong absorption of a proportion of the incident light by free ligand (TPP) before it can excite the sample (‘primary’ IFE), and which therefore decreases the observed fluorescence. The correction factor was determined empirically from the fluorescence intensity of free TPP in 50 mM Tris-HCl buffer as described by MacDonald *et al*.^33^ (Fig. S2, Supplementary Information).

### Characterisation of TPP binding to wild–type *E. coli* transketolase

Since the original detection of TPP binding in the absence of divalent cations^26,27^ there have been no reports of the associated binding parameters. We therefore determined these, in addition to carrying out the first comprehensive study of TPP binding over large cofactor concentration ranges, for purified *E. coli* transketolase.

**Figure 2:**
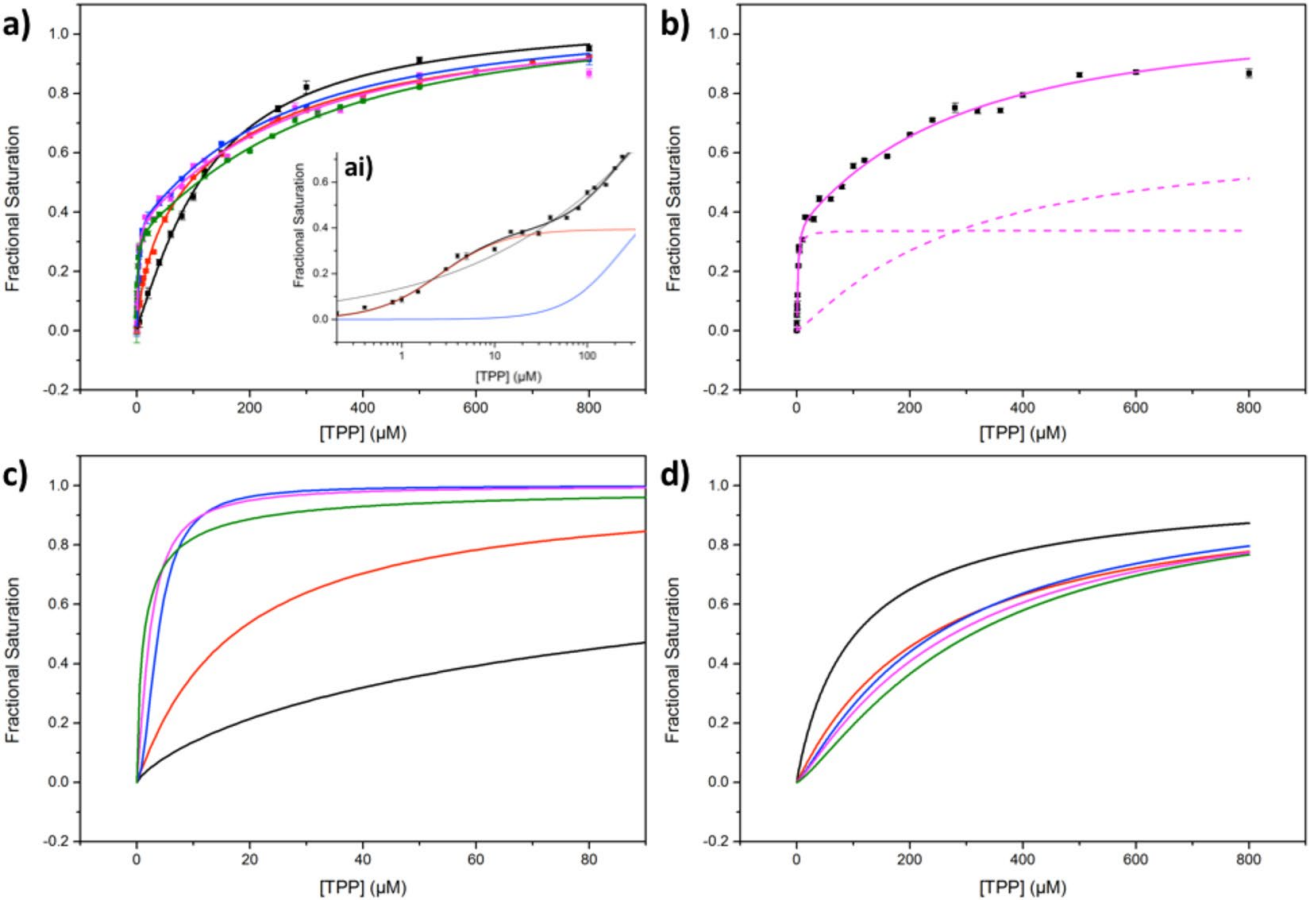
Experimental data of 0.05 mg/mL wild-type TK binding to TPP at 0 mM (black), 1 mM (red), 4.5 (blue), 9 mM (magenta) and 18 mM (green) Mg^2+^. Experimental data-points (**a**) at all [Mg^2+^] fitted to the double-Hill function; ai) at 9 mM Mg^2+^ plotted on a logarithmic × axis and fitted to a single Hill function (thin black) and the double-Hill function (thick black) to show the superior fit to the latter function. The contributions of the high (red) and low affinity (blue) binding sites of the double-Hill function are also shown; (**b**) at 9 mM Mg^2+^ (double-Hill function) with the contributions of the high and low affinity binding sites shown as dashed lines; normalised contributions to double-Hill functions, of the (**c**) high affinity and (**d**) low affinity binding sites at each [Mg^2+^]. For reference, the data in (**b**–**d**) were derived from the experimental data and corresponding fits plotted in (**a**).

The form of the binding isotherm of TPP with TK was found to be dependent on [Mg^2+^], and indicated a shift in binding cooperativity at higher [Mg^2+^] (Fig. 2a–d). The weighted sum of two Hill functions (the double-Hill function; Section 3, Supplementary Information), describing two independent binding events, both cooperative in nature, was found to give a significantly better fit to the TPP-binding isotherms compared to a standard Hill function (Figs 2ai; S3, Supplementary Information) and was thus used to determine the TPP binding parameters at various [Mg^2+^] from 0–18 mM. This identified two dissociation constants *K*_*d(high)*_ and *K*_*d(low)*_, which were independent from each other, reflecting two distinct populations, TK_high_ and TK_low_, as described below. Figure 2b provides an example of the weighted contributions of TPP binding to TK_high_ and TK_low_ (dashed magenta lines) that sum to give the overall fit to the double-Hill function (solid magenta line) at 9 mM Mg^2+^. To visualise and compare the individual Hill functions derived from the double-Hill function, which described TPP binding to TK_high_ (Fig. 2c) and TK_low_ (Fig. 2d) between 0–18 mM Mg^2+^, each weighted contribution was normalised to one.

#### Binding affinity of TK_high_

The fluorescence quenching data revealed a high-affinity TPP-binding event with a dissociation constant, *K*_*d(high)*_, of 113 ± 40 µM (Fig. 2; Table 1) at 0 mM Mg^2+^, consistent with the only comparable values available in the literature for *E. coli* transketolase of *K*_*d*_ = 29 µM and 8 µM at 0.01 mM and 0.1 mM Mg^2+^, respectively), which were obtained indirectly from kinetic enzyme activity data^34^.

**Table 1:** Summary of the binding parameters of the high affinity binding site, TK_high_, when fitted to the double-Hill function.

[Mg^2+^] (mM)	*K*_*d(*high)_ (µM)	±	*n*_*(high)*_	±
0	113	40	0.67	0.30
1	20.6	4.2	1.01	0.14
4.5	3.79	0.38	1.98	0.38
9	2.29	0.23	1.36	0.16
18	1.31	0.27	0.76	0.06

A TK concentration of 0.05 mg/mL was used in each binding assay. Associated errors are the fitting error for the double-Hill function.

**Figure 3:**
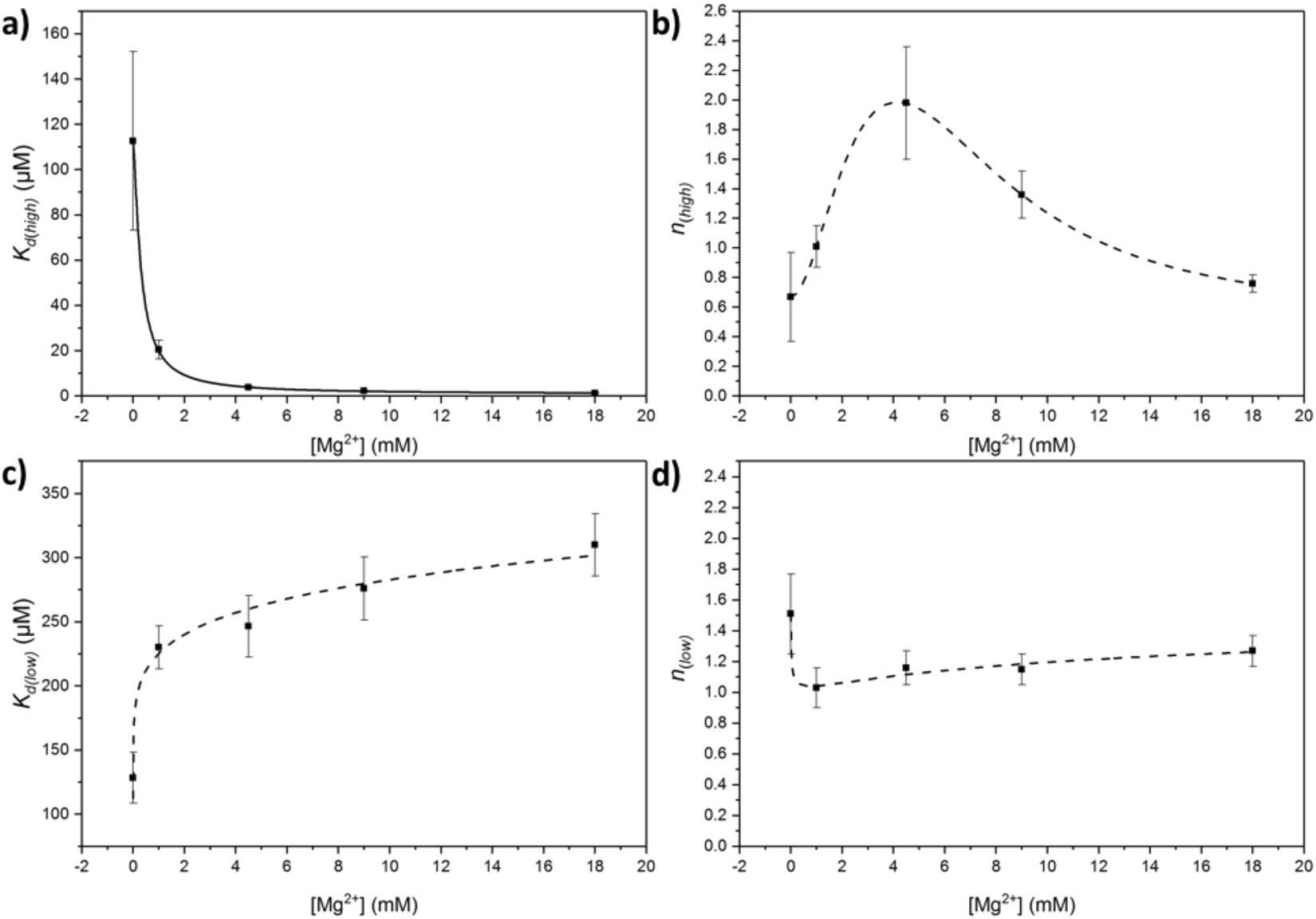
Dependence of TPP-dissociation constants and Hill coefficients, of the high (TK_high_) and low (TK_low_) affinity binding sites for 0.05 mg/mLTK, at 0 mM, 1 mM, 4.5 mM, 9 mM and 18 mM Mg^2+^. (**a**,**b**) the dissociation constants and Hill coefficients of the high (TK_high_) affinity binding site, respectively. (**c**,**d**) - the dissociation constants and Hill coefficients of the low (TK_low_) affinity binding site, respectively. Solid lines represent quantified, fitted graphs; dashed lines represent clear, unquantified trends.

The affinity of TK_high_ for TPP improved markedly in the presence of Mg^2+^, with an 86-fold decrease in the dissociation constant at 18 mM Mg^2+^ relative to that in the absence of Mg^2+^ (Fig. 2; Table 1). By fitting the obtained dissociation constants to a Hill function (Fig. 3a), the dissociation constant at fully saturating [Mg^2+^] was estimated to be 0.93 ± 0.27 µM, a 121-fold improvement compared to that in the absence of Mg^2+^. The largest decrease in *K*_*d(high)*_ was observed at low Mg^2+^ concentrations < 1 mM, as noted by Kochetov *et al*.^26^. The dependence of *K*_*d(high)*_ on [Mg^2+^] fitted to a Hill function with *n* = 1.33 ± 0.14, implying that TK_high_ binds to the two Mg^2+^ ions with slightly positive cooperativity between the two Mg^2+^ binding sites.

#### Binding cooperativity of TK_high_

In the absence of Mg^2+^, the Hill coefficient, an indicator of the degree of cooperativity, suggested negative cooperativity between TPP binding sites (*n*_*high*_ = 0.69 ± 0.30), implying that in the absence of Mg^2+^, binding of the first TPP molecule reduces affinity for the second TPP molecule (Fig. 3b).

The presence of Mg^2+^ ions at low concentrations appears to increase the cooperativity of TPP binding. However, at increasingly high [Mg^2+^] > 4 mM, the Hill coefficient of TPP binding shifts from exhibiting strong positive-cooperativity, with an theoretical total of two TPP molecules binding, back to a negative cooperativity (Fig. 3b; Table 1).

The complex dependence of cooperativity on [Mg^2+^] thus explains why there was no consensus view previously, as the earlier studies each used different Mg^2+^ concentrations. However, a collective review of the studies on yeast transketolase that published a Hill coefficient suggested a similar trend, albeit at lower [Mg^2+^] given the lower intracellular free [Mg^2+^] of yeast relative to *E. coli*^35^: strong positive cooperativity (*n ≈* 2) at 1 mM Mg^2+^ ^29^, slight negative cooperativity at 2 mM Mg^2+^ ^19^ and strong negative cooperativity (*n* = 0.61) at 3 mM Mg^2+^ ^7^.

The molecular basis of the trend reversal at [Mg^2+^] > 4 mM is unclear but may reflect the opposing effects of binding the first and second Mg^2+^ ions on TPP binding. While the binding of the first Mg^2+^ ion may result in cooperative binding of two TPP molecules and significantly improve affinity, binding of a second Mg^2+^ may remove the positive interaction between the first and second TPP-binding sites, and hence introduce negative cooperativity between active sites. It is likely that both TPP binding sites are occupied, even at high [Mg^2+^], because a similar maximum change in raw fluorescence signal was observed across all [Mg^2+^]. It therefore follows that at higher [Mg^2+^] the apparent dissociation constants may in fact be a convolution of the binding parameters of two non-equivalent sites. Given the sensitivity of fluorescence measurements, it may be possible to concentrate data points over a much smaller range at low TPP concentrations and deconvolve the two separate dissociation constants.

### Detection and characterisation of a novel low-affinity species, TK_low_

The fluorescence quenching data fitted best to the weighted sum of two Hill functions (the double-Hill function; Section 3, Supplementary Information) that described two independent, multi-ligand binding events; one with a high affinity dissociation constant (described above) and one with a low affinity dissociation constant (Fig. 2d; Table 2). This low affinity TPP-binding event has never been previously characterised in *E. coli* nor *S. cerevisiae* transketolase. As transketolase exists as both monomer and dimer, depending on [TPP] and [TK], and assuming that TK_high_ and TK_low_ forms pre-exist as monomers, then three dimeric species are, in theory, possible: TK_high_-TK_high_, TK_low_-TK_low_ and the mixed dimer species, TK_high_-TK_low_. In this scenario, the relative populations of TK_high_ and TK_low_ as monomers, homo-, and hetero-dimers prior to TPP-binding, may potentially impact the apparent dissociation constants and Hill coefficients of TK_high_ and TK_low_ subunits. The double-Hill function used to fit the data made no assumptions regarding the conformational arrangements of TK_high_ and TK_low_, and so the derived binding parameters are a convolution of the true binding parameters of TK_high_ and TK_low_ within each conformational arrangement. We attempted to fit the data to a triple-Hill function, but a third binding event could not be resolved. It could be that TK_high_ and TK_low_ monomers each only self-associate, and do not form a mixed dimer. However, the resolution of only two sets of binding parameters does not rule out the existence of a third, representing the mixed dimer species, as each sub-unit within a mixed dimer may simply have identical or near-identical binding parameters to those within one of the homodimers.

**Table 2:** Summary of the binding parameters of the low affinity binding site, TK_low_, when fitted to the double-Hill function.

[Mg^2+^] (mM)	*K*_*d*(low)_ (µM)	±	*n*_*(low)*_	±
0	129	20	1.51	0.26
1	230	17	1.03	0.13
4.5	247	24	1.16	0.11
9	276	25	1.15	0.10
18	310	24	1.27	0.10

A TK concentration of 0.05 mg/mL was used in each binding assay. Associated errors are the fitting error for the double-Hill function.

#### Binding affinity of TK_low_

Interestingly, the addition of Mg^2+^ to TK_low_ had the opposite effect on TPP binding compared to TK_high_. The presence of Mg^2+^ decreased the affinity of TK_low_ for TPP, although to a much lesser degree, with a 2.4-fold increase in *K*_*d(low)*_ as [Mg^2+^] increased from 0 mM to 18 mM (Fig. 3c; Table 2). Although the dissociation constant clearly increased with [Mg^2+^], it was difficult to define anything other than the general trend when fitted to a Hill function.

#### Binding cooperativity of TK_low_

The trend in the binding cooperativity of TK_low_ was also the inverse of TK_high_. At low [Mg^2+^], *n*_*low*_ initially decreased to 1 and hence removed cooperativity between active sites; above 2 mM Mg^2+^ *n*_*low*_ increased slightly but not significantly (Fig. 3d; Table 2). Generally, the cooperativity of binding to TK_low_ was less cooperative than TK_high_, which may indicate a disruption in the cross-talk and connectivity between active sites in the TK_low_ dimer, potentially through disturbance of the proton wire.

#### The ratio of [TK_high_]:[TK_low_] was invariant to [Mg^2+^]

By fitting the data to the double-Hill function, we could determine the ratio of TPP binding to TK_high_ and TK_low_ (*B*_*max*_ ratio), which reflected the ratio of [TK_high_]:[TK_low_]. For clarity, henceforth %*B*_*max(high)*_ will represent the percentage of all TK that is TK_high_. The proportion of fluorescence quenching attributed to the high-affinity event was independent of [Mg^2+^] (Tables 1 and 2). In addition, the global %*B*_*max(high)*_, derived from a global fit of all TPP-binding data-sets to the double-Hill function with the *B*_*max*_ parameter shared, was 33.6 ± 2.9% over all [Mg^2+^].

#### %B_max(high)_ was invariant to [TK] and the apoTK monomer-dimer equilibrium

**Figure 4:**
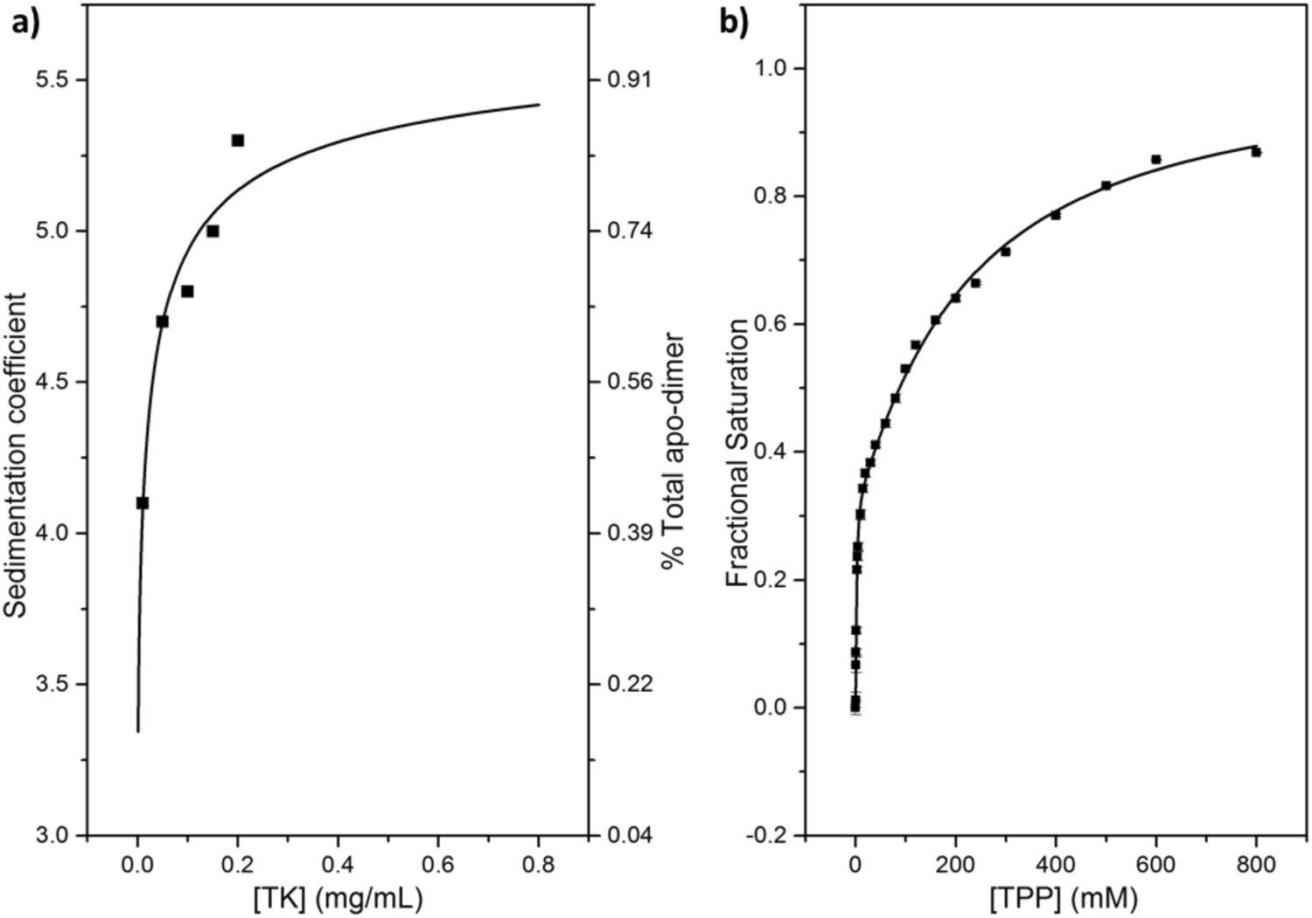
Comparison of (**a**) apo-dimer formation as a function of [apoTK] and (**b**) TPP-binding at 0.2 mg/mL. (**a**) The change in sedimentation coefficient, S_w,20_, which is equivalent to the change in dimer fraction (right axis), at pH 7.0 as a function [apo-TK]. Experimental data was fitted to the weighted sum of two dimerization functions^45^ describing a two-dimer system comprised of TK_high_-TK_high_ and TK_low_-TK_low_, as described. (**b**) Experimental data of 0.2 mg/mL wild-type TK binding to TPP at 9 mM Mg^2+^, fitted to a double-Hill function.

Analytical ultracentrifugation (AUC) data indicated that an increase in [TK] shifted the equilibrium between the monomeric and dimeric forms of apoTK towards dimeric apoTK (Fig. 4; S4, Supplementary Information). An increase in [TK] from 0.05 mg/mL, the TK concentration in the previous binding assays, to 0.2 mg/mL, increased the fraction of the initial apoTK dimer from 62.4% to 83.4%. In contrast, when the TPP-binding assay was repeated at 0.2 mg/mL, the *%B*_*max(high)*_ was 32.9 ± 1.5% (Fig. 4) compared to 33.6 ± 2.9% at 0.05 mg/mL TK, and hence the *%B*_*max(high)*_ was invariant to both [TK] and the resulting shift in the monomer-dimer equilibrium prior to TPP-binding. Therefore, the relative populations of TK_high_ and TK_low_ as measured in the final homodimer forms were not influenced by the monomer to dimer equilibrium prior to TPP binding, but resulted directly from a pre-existing population of TK_high_ and TK_low_ subunits.

### TK_high_ and TK_low_ are two distinct forms of transketolase

The co-existence of two distinct forms of TK, with different affinities for TPP, but with no previous structural evidence to suggest how they differ, prompted us to look for chemical changes by mass spectrometry.

#### Detection of transketolase post-translational modification by mass spectrometry

**Figure 5:**
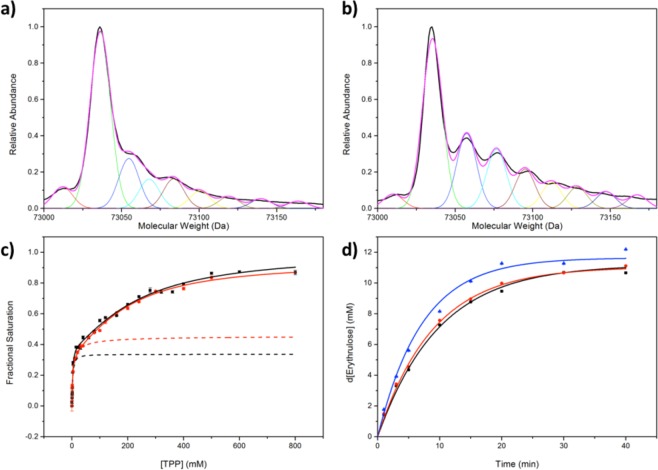
The mass spectra of purified wild-type transketolase expressed in (**a**) the absence and (**b**) the presence of 0.005% CHP added half-way through an eight-hour fermentation, fitted to the sum of multiple Gaussian functions. The major peak (green) corresponds to unmodified transketolase, while the next two peaks (blue and cyan) correspond to modified transketolase. Higher molecular-weight peaks correspond to inactive, over-oxidised TK. (**c**) Experimental TPP-binding data of 0.05 mg/mL wild-type TK expressed in the presence (black) and absence (red) of 0.005% CHP, with 9 mM Mg^2+^, fitted to a double-Hill function. Dotted lines represent the contribution from TK_high_ and hence the *%B*_*max(high)*_. (**d**) Activity data of purified 0.067 mg/mL wild-type transketolase; black: 50 µM TPP, 9 mM Mg^2+^; red: 2.4 mM TPP, 9 mM Mg^2+^; blue: wild-type TK expressed the presence of 0.005% CHP, with 2.4 mM TPP, 9 mM Mg^2+^.

Liquid chromatography electrospray ionisation mass spectrometry (LC-ESI-MS) of purified wild-type transketolase revealed a major unmodified transketolase species at the predicted molecular weight of 73,035 Da, and two higher molecular weight species with smaller peak areas (Fig. 5a). At even higher molecular weights, other yet smaller peaks were present, though most were not well resolved. The peaks were fitted to the sum of multiple Gaussian functions to determine the peak area and hence abundance of the unmodified transketolase species, TK_unmodified_, relative to the first two, best defined, higher molecular weight species, defined as TK_modified_ (i.e. peak two and three combined). The %TK_modified_ was 31.0 ± 1.7%, comparable to the %TK_high_ (33.6 ± 2.9%) obtained from the global %*B*_*max(high)*_ (Tables 1 and 2), implying that TK_low_ was the unmodified transketolase species, whilst TK_high_ formed as the result of post-translational oxidation during fermentation or purification. The average difference in molecular weight between each of the first three transketolase peaks was 15.8 Da, the equivalent of an oxygen atom. Furthermore, over-oxidation of sulfenic acids leads to the formation of sulfinic and sulfonic acids, each leading to further 16 Da increases in molecular weight per oxidation.

The mass spectrometry data alone is insufficient to categorically rule out TK_modified_ as a collection of singly-oxidised species, modified at a number of different residues located near the TPP-binding site (e.g. Met153, Cys157, Met158, Met159, Cys167). Equally, the more-oxidised, inactive species with the highest molecular weight peaks are potentially a combination of over-oxidised states at these residues. We therefore attempted to pinpoint the oxidation modification by re-analysis of previously-published X-ray crystal structures.

#### Crystallographic evidence of sulfenylation at Cys157

It was indicated previously from X-ray crystal structure data^9^ that Cys157, located near the TPP-binding site, was present in an unusual sulfenic acid form, which would have an increased molecular weight of 16 Da relative to unmodified cysteine^36^. Later work found that direct air-oxidation *in vitro* was deactivating for TK, though only in the presence of TPP^36^, and the authors suggested that the crystallographic evidence for a sulfenylated Cys157 may have been an artefact of the crystallization process. Our mass spectrometry data indicated that in fact the sulfenylation of Cys157 was not an artefact of crystallization, but rather occurred during expression or purification of the enzyme. Interestingly, we found that at least one of the oxidised states was more active than the unmodified TK, which means that the inactivated product of air-oxidation observed previously by Mitra *et al*.^36^, was not TK_high_, and that the two oxidation mechanisms are different, or perhaps that air-oxidation led to lower activity through over-oxidation at the same site.

**Figure 6:**
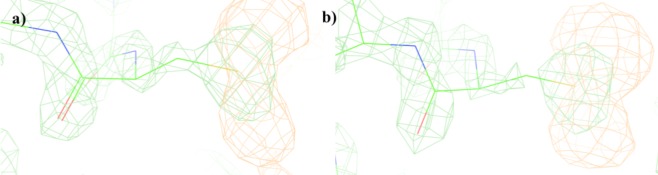
The 2F_0_F_C_ (green) and F_0_F_C_ (orange) electron density difference maps around residue Cys157 in subunit A and B of wild-type *E. coli* TK (1QGD) at 3σ omit level. The 2F_0_F_C_ maps illustrate the fitted electron density of the solved structural model. The F_0_F_C_ maps illustrate the electron density that has not been accounted for in the structural model.

The detection of transketolase oxidation via mass spectrometry prompted us to re-inspect the published electron density maps for the crystal structure of wild-type *E. coli* holo-transketolase (1QGD). In the published structure, Cys157 was retained as unmodified sulfhydryl. However, closer inspection of the 2F_0_F_C_ and F_0_F_C_ electron density difference maps around residue Cys157 in subunit A and B, at 3σ omit level (Fig. 6a,b), suggested a substantial amount of electron density that has not been accounted for in the structural model. This difference in electron density can be explained by the existence of populations of singly- and doubly-oxidised Cys157 to form the sulfenic acid and sulfinic acid, respectively, as also detected by LC-ESI-MS.

Cys157 is conserved across many bacteria, yeast, protozoa and plants but not in animal species such as *Homo sapiens* or *Mus musculus*. It is therefore likely that many domains of life upregulate transketolase via oxidation of Cys157.

#### Oxidative stress during fermentation increased %TK_modified_, %TK_high_ and enzyme activity

The effect of increasing cellular oxidative stress on the %TK_modified_, %*B*_*max(high)*_ and specific activity was investigated by addition of 0.005% cumene hydroperoxide (CHP) to *E. coli* cells half-way through an eight-hour fermentation. LC-ESI-MS, TPP-binding and specific activity data revealed a proportional increase in the %TK_modified_, %*B*_*max(high)*_ and activity by approximately 37% (Fig. 5b–d; Table 3). These findings not only confirm that TK_low_ and TK_high_ are equivalent to TK_unmodified_ and TK_modified_, respectively, but provides a strong causative link where the more active TK_high_ is formed as a direct response to oxidative stress during fermentation.

**Table 3:** Summary of the *%B*_*max(high)*_, % TK_modified_ and specific activity of wild-type transketolase in the presence and absence of 0.005% CHP.

TK variant	*%B*_*max(high)*_	±	%TK_modified_	±	Specific activity (μmol/mg/min)	±
Wild-type	33.6%	2.9%	31.0%	1.7%	0.31	0.01
Wild-type ± 0.005% CHP	45.8%	3.8%	44.1%	1.5%	0.41	0.01

Associated errors are the fitting error when fitting to the respective functions.

We also examined the addition of reducing agent (10 mM β-mercaptoethanol) during the lysis and purification of TK from cells grown in the absence of CHP (Fig. S5, Supplementary Information). This had no impact on %TK_modified_ as determined by LC-ESI-MS, which strongly indicated that the oxidation of TK_low_ (TK_unmodified_) to TK_high_ (TK_modified_) occurred intracellularly during fermentation rather than during the lysis and/or purification steps. However, the reducing agent decreased the proportion of over-oxidised transketolase species. This is consistent with a separate oxidation pathway due to air-exposure after the fermentation, that leads to the enzyme inactivation observed previously.

#### Detection and location of sulfenic acid formation via dimedone labelling

**Figure 7:**
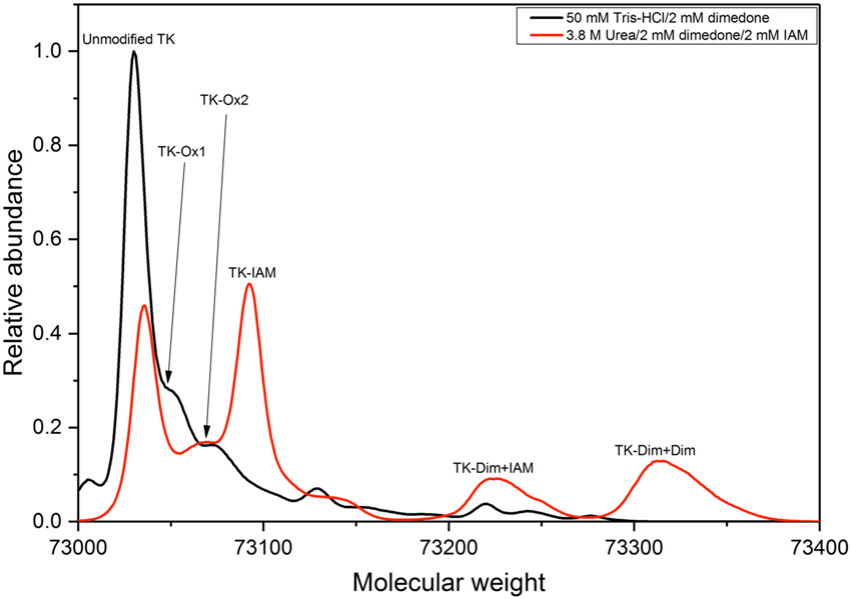
Evidence for the existence of sulfinic acid modifications. Intact mass spectra of wild-type transketolase reacted with 2 mM dimedone in the absence (black) and presence (red) of 3.8 M urea and 2 mM iodoacetamide (IAM), normalised to the total integrated peak area. The spectra show the shift in molecular weight as a result of reaction between dimedone molecules and protein sulfinic acids. -Ox1 corresponds to one sulfinic acid modification; -Ox2 corresponds to two sulfinic acids or one sulfonic acid modification; -IAM corresponds to carbamidomethylation; and –Dim corresponds to dimedone modification at sulfinic acids.

Dimedone specifically reacts with sulfenic acids, but not with thiol groups or their over-oxidised states such as sulfones, and was hence used to detect the presence of sulfenic acid(s) through intact protein LC-ESI-MS. Apo-dimeric wild-type transketolase was initially reacted with dimedone in 50 mM Tris-HCl. No shift in molecular weight was observed, presumably because any sulfenic acids were not sufficiently solvent-exposed in the correctly-folded apo-dimeric transketolase (Fig. 7). To increase the solvent accessibility of any sulfenic acids, the reaction was repeated in 3.8 M urea, the highest concentration transketolase can tolerate without causing irreversible denaturation^37^, with iodoacetamide to trap any exposed sulfhydryls, and minimise their oxidation into sulfenic acids. A 53% decrease in the relative unmodified peak area, and the equivalent area for a newly formed carbamidomethylated peak, corresponded to iodoacetamide labelling of an exposed sulfhydryl (Fig. 7). Two new peaks were formed, corresponding to one and two dimedone-labelled sulfenic acids. The first was also carbamidomethylated, suggesting it simultaneously contained the labelled sulfhydryl. The second only contained the two dimedone labels. Dimedone labelling was accompanied by a significant decrease in relative peak area for the singly-oxidised ( + 16 Da) wild-type transketolase, which thus corresponded to a sulfenic acid of TK_high_. By contrast, the second, doubly-oxidised relative peak area ( + 32 Da), which corresponded to two sulfenic acids or one sulfinic acid modification per TK molecule, remained unchanged, presumably because dimedone labelling of the former approximately equalled sulfone formation from sulfenic acids that escaped dimedone labelling in the presence of urea.

No peak-shift was observed that corresponded to the reaction with two dimedone and one iodoacetamide molecules, which implied the peak that corresponded to a reaction with two dimedone molecules may in fact be an artefact from oxidation of a sulfhydryl to a sulfenic acid, which was subsequently trapped by a dimedone molecule.

### TK_low_ is a low-activity form of transketolase

The activity of wild-type transketolase towards 50 mM glycolaldehyde (GA) and 50 mM HPA at 2.4 mM TPP and 9 mM Mg^2+^ (TK_high_ and TK_low_ saturated) increased only 9.0% relative to the activity at 50 µM TPP (only TK_high_ saturated) (Fig. 5d). This was significantly less than the expected increase of approximately 230% if the holoTK_low_ activity was equal to that of holoTK_high_. The activity data were combined with the fractional saturation of TK_high_ (98.5% and 100%) and TK_low_ (12.2% and 92.4%) from the TPP binding data at 9 mM Mg^2+^ and 50 µM or 2.4 mM TPP, respectively, and the activity of each species that contributed to the total observed was calculated using a pair of simultaneous equations. The solution to these equations revealed that the activity of TK_low_ towards GA was only 4.5% relative to that of TK_high_. Therefore, the TK_low_ dimer was effectively inactive, and the oxidation of TK to form TK_high_ resulted independently in increased dimer formation, higher affinity for TPP, and also inherently higher activity.

### Ruling out alternative possible origins of %B_max(high)_

While the mass differences correlated directly to the observed ratio of TK_high_:TK_low_ from the %*B*_*max(high)*_ ratio, several other potential mechanisms might be hypothesised to explain the observed TPP-binding behaviour. These include: i) allostery; ii) asymmetric TPP binding to non-identical active sites in all homodimers; iii) population of intermediate states during TPP binding. However, none of these mechanisms fit the observations made in our work, and we have addressed each of them in detail in the Supplementary Information (Section 6). We also examined whether a lack of TPP during overexpression might provide the conditions under which TK differentiates into the TK_high_ and TK_low_ forms. *E. coli* is known to form approximately 6% holo-TK from overexpressed TK, when reliant on only the available cellularly synthesised TPP^38^. However, supplementation of the fermentation culture with 0.5 mM thiamine, which *E. coli* can import and subsequently convert to TPP, was found to have no significant effect on the %*B*_*max(high)*_ obtained (Fig. S6, Supplementary Information).

### The two-species model of transketolase activation

**Figure 8:**
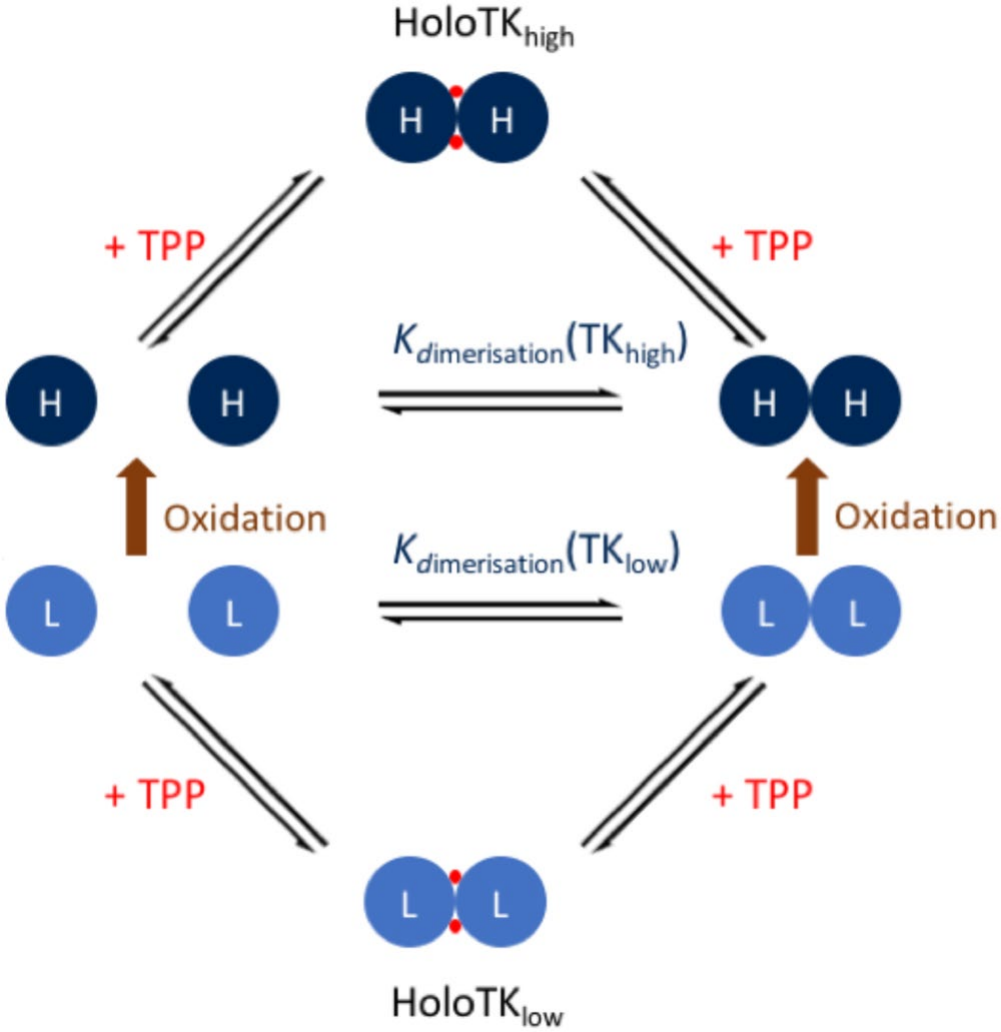
A schematic diagram of the two-species model of TK activation. The model is based on the combined TPP-binding, AUC, mass spectrometry and enzyme activity data. L (light blue) represents a TK_low_ monomer and H (navy blue) a TK_high_ monomer. TK_low_ is converted to TK_high_ via oxidation (brown) of an active site methionine or cysteine. The most likely modification site is Cys157.

Finally, we propose the Two-Species Model of transketolase activation (Fig. 8), which describes TPP-binding to TK_high_ and TK_low_, and the conversion of inactive TK_low_ to active TK_high_ via oxidation of Cys157.

## Discussion

It is unclear why TK_low_ was not detected in other cofactor-binding studies, but this could have been one, some or all of the following reasons: (a) the larger range of cofactor concentrations used in this study; (b) the larger datasets taken at each [Mg^2+^]; (c) there may be no inducible absorption band at 320 nm upon cofactor binding to TK_low_; and (d) TK_low_ activity is negligible relative to TK_high_ and so invisible to measurements of cofactor affinity based on activity measurements. The presence of a low-affinity transketolase binding event has been suggested previously for yeast transketolase^29^ but was not further characterised. Therefore, for decades it has been assumed that transketolase expressed and assembled always as a single native conformation, when in fact two monomeric species may often have been formed. Crystallography studies would not have revealed the second species simply through partial TPP occupancy as cofactors have always been added to saturation, even of the low-affinity site^8,9,22,23^.

The available crystal structures of transketolase have not been able to reveal a structural basis for the non-equivalence of the two active sites in terms of TPP-binding cooperativity, or alternating sites reactivity. In the one case where this has been observed in a TPP-dependent enzyme, it manifested as subtle differences in the mobility of structural regions around the active site of E1 PDC^21^. Such differences are difficult to observe, are potentially subdued by crystal packing, and could readily average out rather than resolving into distinct conformers. Furthermore, non-equivalence may originate from dynamics, rather than underlying structural differences, between the active sites and would therefore only be detected in kinetic-based assays. Similar differences between TK_high_ and TK_low_ would also be challenging to resolve. Even the structural differences resulting from the addition of only one or more oxygen atoms as detected by mass spectrometry, would be difficult to resolve, or easily overlooked by crystallography. Additionally, the oxidation state and hence formation of TK_high_ may yet be found to vary considerably with the range of expression systems and bioreactor conditions used in different studies, as these would lead to different levels of oxidative stress *in vivo*. Thus, many crystal structures and studies of TK may have been carried out on samples with much lower levels of TK_high_.

The detection of TK_low_ also highlights a major challenge with measuring the specific activity or *k*_*cat*_ of overexpressed enzymes. It has always been assumed that saturation with cofactors led to maximum activity. However, the TK_low_ species accounts for roughly two-thirds of the purified transketolase in solution, but only 9% of the overall catalytic activity. Therefore, the true catalytic potential, in particular *k*_*cat*_ for the active transketolase species, TK_high_, has been wildly underestimated. Our research also showed that the standard concentrations of 2.4 mM TPP and 9 mM Mg^2+^ are unnecessarily high for studying TK_high_, but have been likely optimised to achieve maximum activity through cofactor-saturation, in samples that were predominantly or even entirely the un-oxidised TK_low_ form. Going forward, industrial applications and experimental work may need to re-optimise the fermentation conditions to maximise TK_high_, such as by maximising oxidative stress, and then adopt a working concentration of only 70 µM TPP to give a TK_high_ saturation of 99% at 9 mM Mg^2+^.

The observed oxidation of TK caused a dramatic 100-fold improvement in TPP-affinity and a 20-fold increase in the inherent catalytic activity of the enzyme. Therefore, the oxidation to TK_high_ is potentially also physiologically important, either as a necessary post-translational modification for normal cellular function, or as a rapid response mechanism to cope with the increased metabolic demands under oxidative stress. Metabolically, this is compatible with the location of transketolase in the PPP, and transketolase may indeed act as a redox-sensitive regulatory mechanism that increases metabolic flux through the PPP in times of oxidative stress. Furthermore, the location and potential role of the identified sulfenic acids in the formation of a proton wire between active sites suggests a second potential function of the sulfenic acid. However, while we have shown that induction of oxidative stress during fermentation can lead to an increase in TK_high_, further oxidation during purification cannot be ruled out at this stage. Nevertheless, a 20-fold oxidative-stress induced improvement in activity relative to that under milder oxidative conditions is unusual, and could potentially be exploited deliberately for biocatalysis.

While TK_low_ is unmodified, and assumed in all previous literature to be the physiologically relevant form, it is unclear whether only the overexpression in *E. coli* under oxidative conditions designed to maximise cell growth, has led to the partial oxidation to form TK_high_, or whether or not this form exists naturally. It is known that air-oxidation of native holo-TK actually leads to loss of activity via an unknown mechanism, but one that must be either at a different site^39^, or resulting from over-oxidation at the same site. Therefore, the activating oxidation in TK_high_ *in vivo* must be more site-selective or controlled, or even occurring only at an intermediate stage of translation and folding.

The complex trends in affinity and cooperativity of TK_high_ binding to TPP and Mg^2+^ are of a form that is often found to be physiologically important. TK_high_ binds TPP with the highest cooperativity at 4 mM Mg^2+^ which matches the intracellular concentration of free Mg^2+^ in *E. coli* of approximately 1–5 mM^35^. Therefore, *E. coli* transketolase has evolved to utilise Mg^2+^ to (a) enhance the affinity of TK for TPP; and (b) maximise cooperativity of binding at physiologically relevant concentrations of Mg^2+^, with the ultimate goal of increasing TK saturation by TPP at lower [TPP]. It is therefore likely that the decrease in cooperativity at > 4 mM Mg^2+^ is not physiologically relevant. Nevertheless, these trends remain acutely relevant to *in vitro* biocatalysis which often pushes enzymes far beyond their natural means, and for which maximizing the activity from overexpressed enzymes is desirable.

Given the potential tripling in activity offered by simply converting TK_low_ to TK_high_, we believe further research into the origin of the two subpopulations is warranted, as would research into methods that can enhance the population that is correctly oxidised to TK_high_, given that this form was the most active. It would also be interesting to analyse donor substrate binding and inhibition, and the unusual phenomenon of heat-activation of transketolase^40^, with respect to TK_high_ and TK_low_.

## Methods

### Materials

TPP, MgCl_2_, glycolaldehyde (GA) and erythrulose [Ery] were purchased from Sigma-Aldrich; Tris-HCl was purchased from VWR International and Guanidine-HCL was purchased from Life Technologies Ltd. HPA was synthesised by reacting bromopyruvic acid with LiOH, as described previously^41^.

### Enzyme preparation

Wild-type transketolase with an N-terminal His6-tag was expressed in *E. coli* XL10-gold cells (Agilent Technologies Ltd) from the plasmid *pQR791*. The resulting cell pellet was lysed and purified as described previously^37^. Purified transketolase was ultrafiltrated four times using Amicon Ultra-4 10k MWCO centrifugal filter to remove excess imidazole and cofactors and subsequently dialysed overnight at 4 °C in 50 mM Tris-HCl, pH 7.0 to obtain apo-TK. Protein concentration was determined by absorbance at 280 nm in 6 M Guanidine-HCl and 20 mM Sodium Phosphate, pH 6.5. Absorbance was measured using a Nanodrop spectrophotometer, assuming a monomeric molecular weight of 73035.5 g mol^−1^ and an extinction coefficient of 92630 L mol^−1^ cm^−1^.

Series of 2x concentrated cofactor solutions were prepared and purified TK was added to a final concentration of either 0.05 mg/mL or 0.2 mg/mL. The samples were incubated at 22 °C for 45 minutes to allow TK-TPP binding to reach equilibrium.

### Fluorescence assay to detect TPP binding

After incubation, the fluorescence intensity of the TK-cofactor samples was determined in a 1.5 mm × 1.5 mm quartz cuvette (Hellma UK Ltd) using a Fluoromax-4 spectrofluorometer (λ_ex_ = 240 nm; λ_ex_ = 330 nm; integration time = 0.1 s; slit width = 8 nm). One measurement per sample was taken, or five measurements per cofactor concentration. The data were corrected for the wavelength dependence of the Xe-lamp intensity and source intensity fluctuations. Inner filter effect (IFE) correction factors (CF) were generated as follows: A stock TPP solution was diluted with 50 mM Tris buffer to generate 5 series of TPP samples between 0 and 1.2 mM TPP. A correction factor was subsequently generated from these data-points by fitting to the function described by MacDonald *et al*.^33^, which accounts for non-linearity in the fluorescence emission. A new correction factor was generated each day the Fluoromax-4 spectrofluorimeter was used.

### Analytical Ultracentrifugation (AUC) Measurements

Analytical ultracentrifugation (AUC) measurements were performed in the Molecular Interaction Facility at UCL by Dr Jayesh Gor. AUC data were obtained for wild-type transketolase in the absence of cofactors Mg^2+^ and TPP at 20 °C on a Beckman XL-1 instrument equipped with AnTi50 rotors. Data were collected at rotor speeds of 40,000 rpm in two-sector cells with column heights of 12 mm. The software SEDFIT was used to analyse the sedimentation data by fitting the experimental interference data using direct boundary Lamm fits of up to 500 scans^42,43^. The resulting size distributions c(s) of oligomers within samples assumed that all species have the same frictional ratio f/f_0_. The c(s) fit was optimised by floating f/f_0_ and the baseline until a sufficiently low root mean square deviation was reached and the visual appearance of the fits were satisfactory. The ratio or percent monomer/dimer within each sample was derived by integrating each peak in the c(s) integration function.

### Transketolase activity assay

Purified, dialysed apo-transketolase (0.2 mg/mL) was incubated with 50 µM or 2.4 mM TPP and 9 mM Mg^2+^ for 45 minutes at 22 °C. 50 µL was added to 100 µL 150 mM GA, 150 mM HPA, giving final substrate concentrations of 50 mM. The reaction was performed in triplicate at 22 °C in a 96 well plate with shaking at 300 rpm using a Thermomixer Comfort shaker. 10 µL of the reaction was quenched with 190 µL 0.1% trifluoroacetic acid (TFA) after 3, 5, 10, 15, 20, 30, and 40 minutes. Samples were subsequently analysed by a Dionex HPLC system (Camberley, UK) with a Bio-Rad Aminex HPX-87H reverse phase column (300 × 7.8 mm^2^) (Bio-Rad Labs., Richmond, CA, USA), via Chromeleon client 6.60 software, to separate and analyse the change in the concentration of substrate (GA) and product (Ery) over the course of the reaction using the method described previously^44^.

### Oxidation of transketolase using cumene hydroperoxide (CHP)

Oxidised samples of transketolase were prepared by the addition of 0.005% CHP to *E. coli* cells half-way through an eight-hour fermentation. *E. coli* cells were subsequently harvested, and transketolase purified and dialysed, as outlined above.

### Mass spectrometry

LC-MS was performed using an Agilent 1100/1200 LC system connected to a 6510 A QTOF mass spectrometer (Agilent, UK). Samples of 10 µL TK at 0.2 µg/µL were injected onto an Agilent PLRP-S (150 mm × 2.1 mm, 1000 Å, 8 µm) column, maintained at 30 °C. Two mobile phases A (5% MeCN in aqueous 0.1% formic acid) and B (95% MeCN, 5% water, 0.1% formic acid) were used at 0.3 mL/min. The column was pre- equilibrated at 25% B for 1.9 min, before injection, held for 1 min further at 25% B, and then a gradient elution increased B to 99% over 16 min. After 2 min, B was decreased to 25% over 0.1 min. The QTOF mass spectrometer scanned m/z from 100 to 3100 Da. Positive electrospray ionisation (ESI) was used with 4000 V capillary voltage, fragmentor at 175 V, skimmer at 65 V and octopole RF peak at 750 V. Nitrogen was used as the nebuliser and desolvation gas at a flow of 5 L/min. Spectra were acquired every second with an acquisition time of 1000 msec/spectrum. Lockspray was used during analysis to maintain mass accuracy. Data were processed in MassHunter software (version B.07.00) and deconvolved using the maximum entropy deconvolution algorithm.

### Dimedone labeling

Apo-transketolase was prepared as described above. 2 mg/mL transketolase was subsequently incubated with 2 mM dimedone in the presence/absence of 3.8 M urea and 2 mM iodoacetamide. The samples were subsequently dialysed overnight at 4 °C in 50 mM Tris-HCl, pH 7.0 to obtain apo-TK labelled with dimedone. Samples were subsequently centrifuged at 15,000 × g for 5 minutes to remove aggregates and prepared for intact protein LC-ESI-MS as described above.

## Supplementary information

Supplementary Information

## Data Availability

The data that support the findings of this study are available from the corresponding author upon reasonable request.
