# Supplementary material for: Novel insights into transketolase activation by cofactor binding identifies two native species subpopulations
Source: Sci Rep. 2019 Nov 6;9:16116. doi: 10.1038/s41598-019-52647-y (PMC6834573; doi:10.1038/s41598-019-52647-y)
Supplement: Supplementary file 1 — Supplementary Information [file 41598_2019_52647_MOESM1_ESM.pdf]

Novel insights into transketolase activation by cofactor binding identifies two native species subpopulations

Henry C. Wilkinson*, Paul A. Dalby

SUPPLEMENTARY INFORMATION

1. Evidence of fluorescence quenching upon cofactor-binding
2. Generation of the inner filter effect correction factor
3. Comparison of experimental TPP-binding data fitted to a single- or double-Hill function
4. Analytical Ultracentrifugation (AUC) interference & c(s) distribution data
5. Lysis and purification in the presence of 10 mM β-mercaptoethanol does not impact $\%B_{max(high)}$
6. Ruling out alternative explanations for the observed $\%B_{max(high)}$ & TPP-binding behaviour
7. Supplementation of fermentation with thiamine

Section 1: *Evidence of fluorescence quenching upon cofactor-binding*

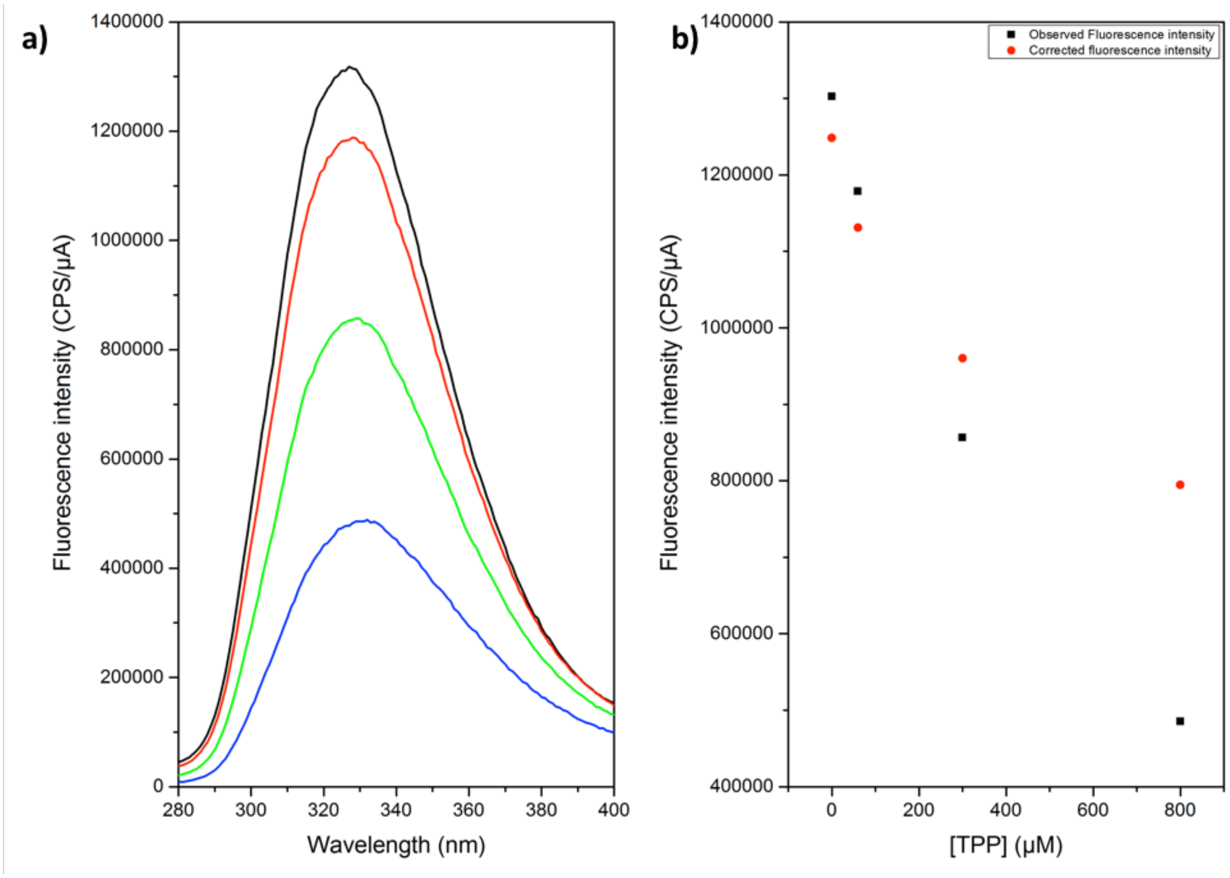

Figure S1: Evidence of fluorescence quenching upon cofactor-binding. a) The observed fluorescence intensity of 0.05 mg/mL TK and 0 (black), 60 (red), 300 (green), and 800 (blue) μM TPP when excited at 240 nm. b) The observed (black) and corrected (red) fluorescence intensity of each sample (λ_{ex} = 240 nm; λ_{em} = 330 nm). Fluorescence intensity was corrected for the inner filter effect (IFE) according to MacDonald *et al*.[1].

Section 2: *Generation of the inner filter effect correction factor*

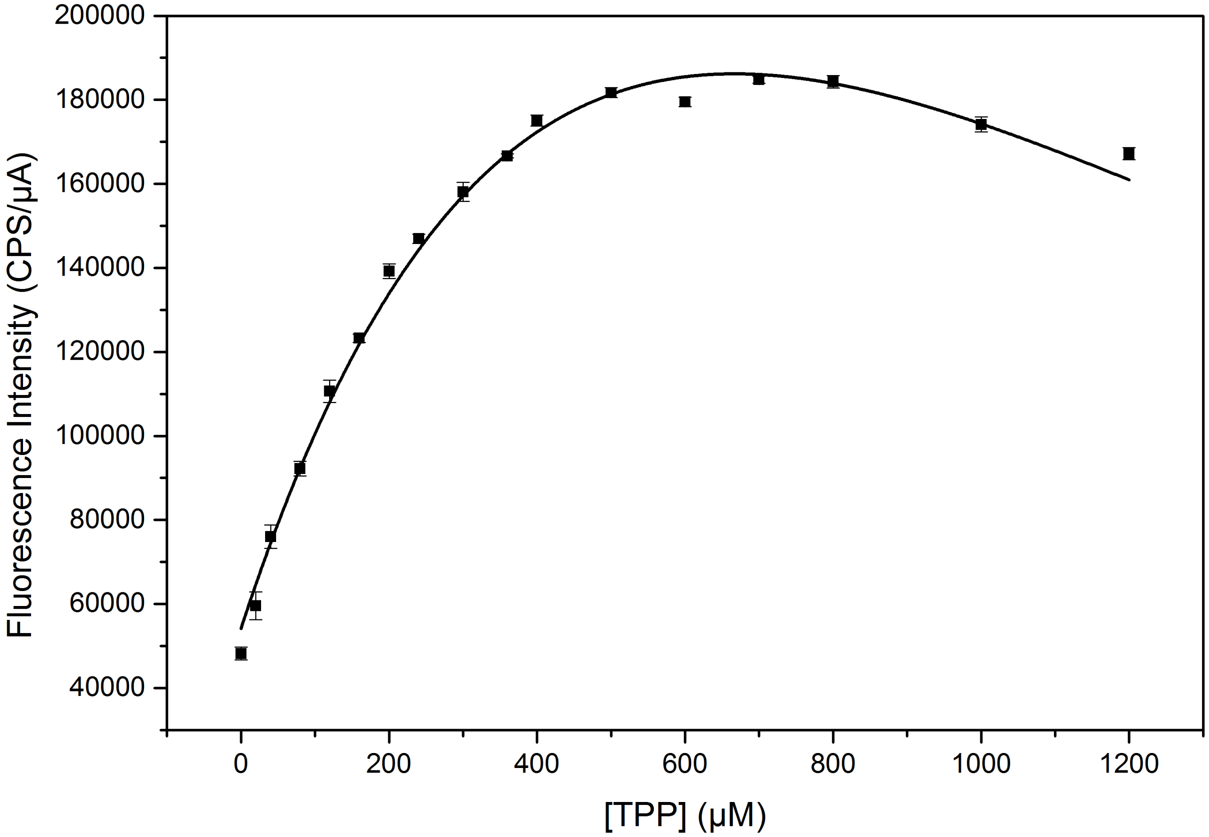

Figure S2: The fluorescence intensity of free TPP in 50 mM Tris buffer (λ_{ex} = 240 nm; λ_{em} = 330 nm), fitted to the correction function described by MacDonald *et al.*[1].

Section 3: *Comparison of experimental TPP-binding data fitted to a single- or double-Hill function*

The double-Hill function: $\theta = \frac{B_{\max(high)} \cdot [L]^{n_{high}}}{K_{d(high)}{}^{n_{high}} + [L]^{n_{high}}} + \frac{\alpha . B_{\max(high)} \cdot [L]^{n_{low}}}{K_{d(low)}{}^{n_{low}} + [L]^{n_{low}}}$

where θ is the fractional saturation, the fraction of [protein] that is bound to ligand; $B_{max(high)}$ is the proportion of TPP that binds to TK_{high}; $\alpha.B_{max(high)}$ is the proportion of TPP that binds to TK_{low}; [L] is the ligand (TPP) concentration; n_{high} and n_{low} are the Hill coefficients of TK_{high} and TK_{low}, respectively; and $K_{d(high)}$ and $K_{d(low)}$ are the dissociation constants of TK_{high} and TK_{low}, respectively.

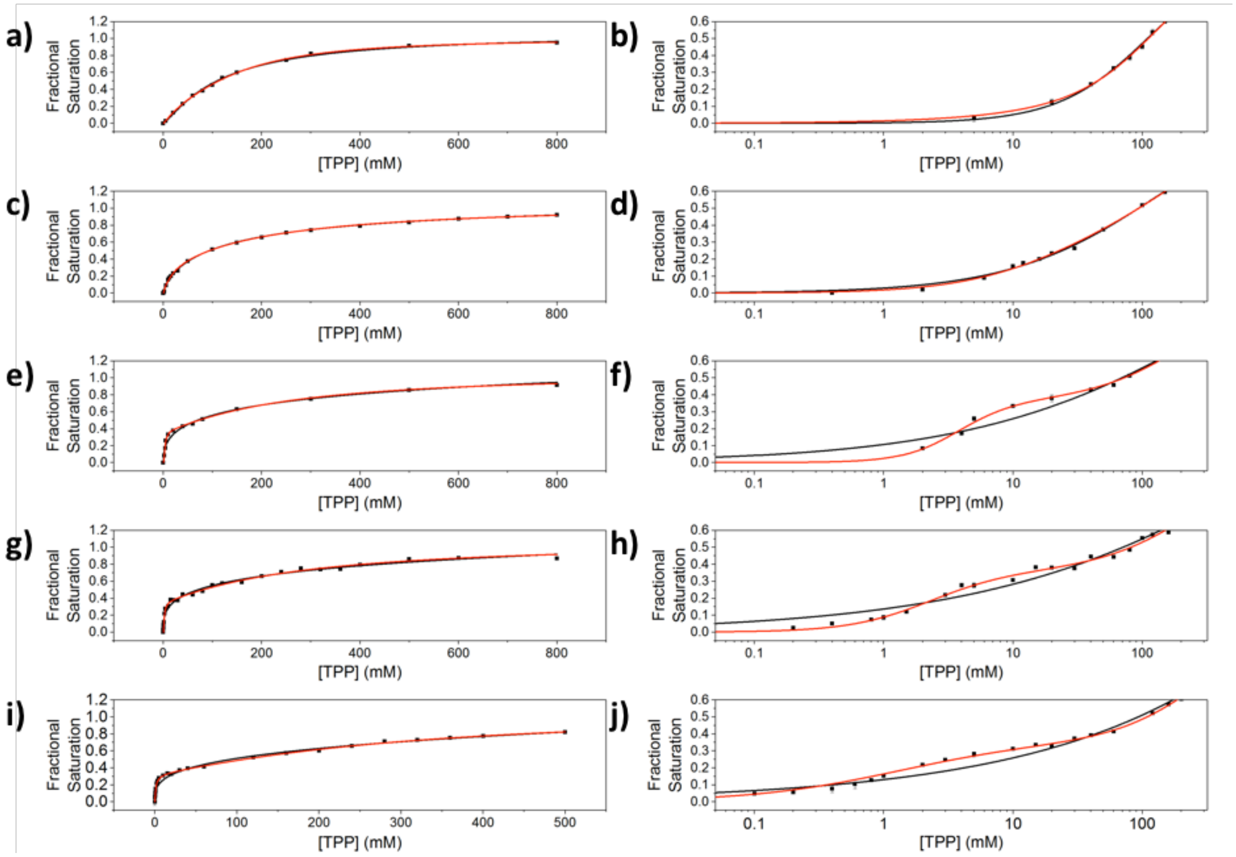

Figure S3: Experimental data of 0.05 mg/mL wild-type transketolase binding to TPP at a) and b) 0 mM; c) and d) 1 mM; e) and f) 4.5 mM; G) and H) 9 mM; and I) and J) 18 mM Mg^{2+}. Experimental data-points were fitted to either a single- (black) or double- (black) Hill function. The data is presented on a logarithmic x-axis to demonstrate the superior fit to the double-Hill function.

The TPP-binding parameters determined using the single Hill (Table S1) have no relatability to previously determined dissociation constants and Hill coefficients; at higher [Mg^{2+}], the fits either had

very large associated errors or the data couldn't be fitted to the single-Hill function. However, those of TK_{high}, determined by fitting the data to the double-Hill function (Table S2), correlated well with previously reported values. We therefore conclude that we have detected two independent binding events, both cooperative, which fitted best to a double-Hill function.

$[Mg^{2+}]$ (mM)	K_d (μM)	±	n	±
0	120	5	1.20	0.04
1	139	9	0.74	0.02
4.5	739	1030	0.42	0.06
9	4390	10100	0.34	0.04
18	-	-	-	-

Table S1: Summary of the TPP-binding parameters when fitted to a single Hill function. A TK concentration of 0.05 mg/mL was used in each binding assay. Associated errors are the fitting error for the single-Hill function. The data couldn't be fitted to the single-Hill function at 18 mM Mg^{2+}.

$[Mg^{2+}]$ (mM)	$K_{d(\mathrm{high})}$ (μM)	±	$n_{(high)}$	±
0	113	40	0.67	0.30
1	20.6	4.2	1.01	0.14
4.5	3.79	0.38	1.98	0.38
9	2.29	0.23	1.36	0.16
18	1.31	0.27	0.76	0.06

Table S2: Summary of the binding parameters of the high affinity binding site, TK_{high}, when fitted to a double Hill function. A TK concentration of 0.05 mg/mL was used in each binding assay. Associated errors are the fitting error for the double-Hill function.

Section 4: Analytical Ultracentrifugation (AUC) interference & c(s) distribution data

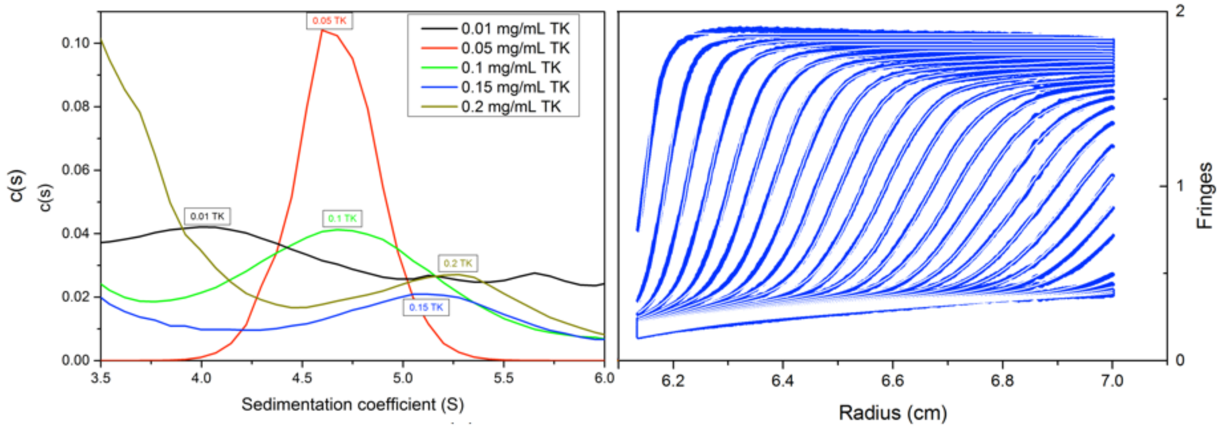

Figure S4: AUC interference data and the derived c(s) distribution and sedimentation coefficients. Plots of c(s) distribution vs sedimentation coefficient at 0.01-0.8 mg/mL TK. The right-hand plot shows the AUC interference fringe data (blue) and best fit (white) from 0.8 mg/mL TK.

Section 5: *Lysis and purification in the presence of 10 mM β-mercaptoethanol does not impact* *%*$B_{max(high)}$

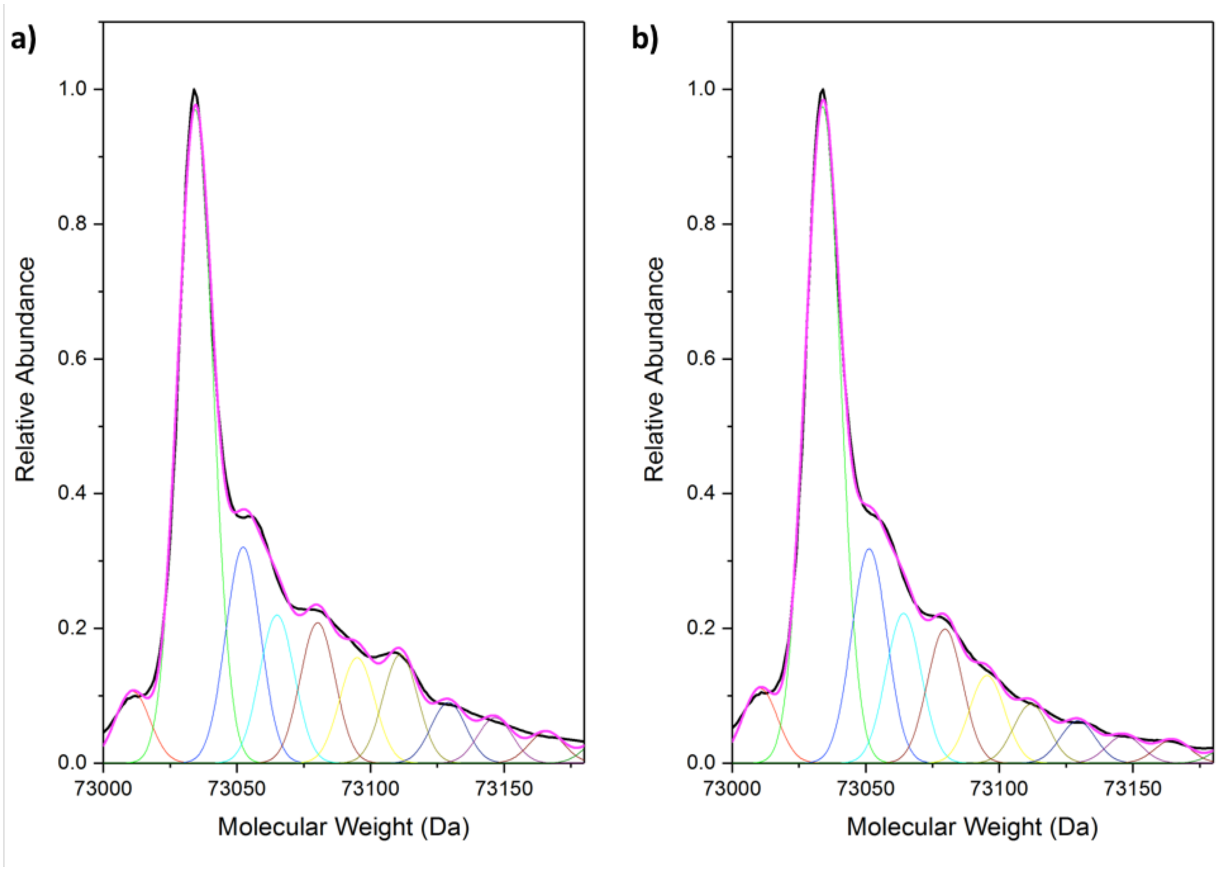

Figure S5: The mass spectra of wild-type transketolase expressed in the absence of β-mercaptoethanol and cumene hydroperoxide, then lysed and purified in the a) absence and b) presence of 10 mM β-mercaptoethanol. The major peak (green) corresponds to unmodified transketolase (TK_{low}), while the next two peaks (blue and cyan) correspond to modified transketolase (collectively TK_{high}). Higher molecular-weight peaks correspond to over-oxidised TK, which are likely inactive.

Section 6: *Ruling out alternative explanations for the observed %$B_{max(high)}$ & TPP-binding*

a) Allostery

Allosteric-activator binding of TPP to transketolase is unlikely given the importance of TPP to the reaction mechanism, and the scarcity of TPP in the cell, and there is no kinetic or structural evidence of such binding outside of the two active-sites. It is therefore likely that the low-affinity binding site is located within the same active-site binding pocket as the high-affinity binding site. Indeed, all X-ray crystal structures of holo-transketolase showed only two TPP molecules per dimer[2,3].

b) Asymmetric TPP binding to non-identical active sites in all homodimers

The presence of two TPP-binding events with significantly different affinities may at first glance support the hypothesis that two TPP molecules bind the two binding sites of a single transketolase dimer asymmetrically; one with high and one with low affinity. This notion is dispelled by several observations. Firstly, we have determined that two independent binding events occur, both cooperative in nature. The observed binding events must therefore be more than cooperativity between only two sites. Furthermore, the dissociation constant of the low-affinity binding site detected in this study is 42-fold and 600-fold greater than those of the two non-equivalent, negatively cooperative active sites reported previously[4], suggesting the low-affinity TPP binding site reported here is an entirely different binding site. Indeed, early work on transketolase cofactor binding detected an additional binding site with a significantly higher K_d than the two non-equivalent 'high-affinity' binding sites detected, but no dissociation constant was obtained, nor was it characterised[5]. The dissociation constant of the high affinity site reported here was between those of the two non-equivalent sites reported previously[4], as would be expected if two dissociation constants are analysed as a single dissociation constant using the Hill function.

c) *Formation of an intermediate state during TPP binding*

Transketolase cofactor-binding and activation has been shown to occur in at least two general steps. The first stage is fast and readily reversible and results in the formation of the catalytically inactive TK---TPP primary complex. Subsequent conformational changes convert the primary complex into catalytically active holo-transketolase, TK*-TPP. The second step is quasi-irreversible and slow in nature[5,6]. In theory, it is possible that TK_{high} and TK_{low} may be synonymous with TK---TPP and TK*-TPP, and that the TK_{low} population is unable to undergo the second, quasi-irreversible step to TK_{high}. However, the maximum TPP concentration used in the previous kinetic experiments using yeast transketolase was 100 μM and therefore only saturating for TK_{high}. As such, the two-step activation of transketolase was observed only in the formation of TK_{high}, and may well be different for TK_{low}. Additional experiments at higher [TPP] would be required to elucidate the mechanism of cofactor-binding and activation of TK_{low}.

Section 7: *Supplementation of fermentation with thiamine*

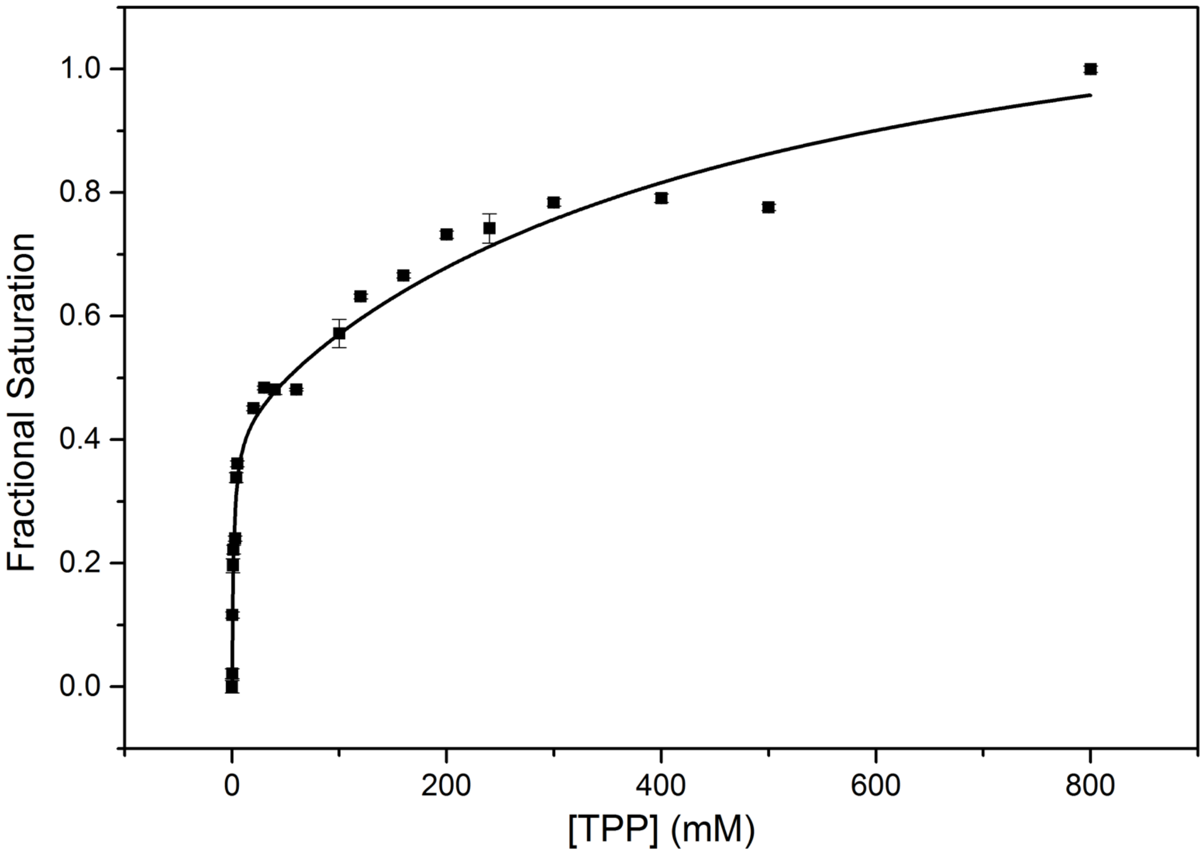

Figure S6: Experimental data of 0.05 mg/mL wild-type transketolase binding to TPP at 9 mM Mg^{2+}, purified from a fermentation supplemented with 0.5 mM thiamine. Experimental data-points were fitted to the double-Hill function (black). The $\%TK_{high}$ was 34.5 + 15.8%, comparable to that of transketolase purified from un-supplemented fermentations.

Supplementary references

1. MacDonald BC, Lvin SJ, Patterson H. Correction of fluorescence inner filter effects and the partitioning of pyrene to dissolved organic carbon. Anal Chim Acta. 1997;338(1–2):155–62.
2. Lindqvist Y, Schneider G, Vihko P. Three-dimensional structure of rat acid phosphatase in complex with L(+)-tartrate. J Biol Chem. 1993;268(28):20744–6.
3. Littlechild J, Turner N, Hobbs G, Lilly M, Rawas A, Watson H. Crystallization and preliminary X-ray crystallographic data with *Escherichia coli* transketolase. Acta Crystallogr Sect D Biol Crystallogr. 1995;51(6):1074–6.
4. Egan RM, Sable HZ. Transketolase kinetics. The slow reconstitution of the holoenzyme is due to rate-limiting dimerization of the subunits. J Biol Chem. 1981;256(10):4877–83.
5. Kochetov GA, Tikhomirova NK, Philippov PP. The binding of thiamine pyrophosphate with transketolase in equilibrium conditions. Biochem Biophys Res Commun. 1975;63(4):924–30.
6. Kovina M V., Selivanov VA, Kochevova N V., Kochetov GA. Kinetic mechanism of active site non-equivalence in transketolase. FEBS Lett. 1997;418(1–2):11–4.
